# MicroRNA Let-7e in the Mouse Prefrontal Cortex Differentiates Restraint-Stress-Resilient Genotypes from Susceptible Genotype

**DOI:** 10.3390/ijms22179439

**Published:** 2021-08-30

**Authors:** Joanna Solich, Magdalena Kolasa, Agata Faron-Górecka, Jacek Hajto, Marcin Piechota, Marta Dziedzicka-Wasylewska

**Affiliations:** 1 Laboratory of Biochemical Pharmacology, Department of Pharmacology, Maj Institute of Pharmacology, Polish Academy of Sciences, Smętna Street 12, 31-343 Kraków, Poland; gaska@if-pan.krakow.pl (M.K.); gorecka@if-pan.krakow.pl (A.F.-G.); wasyl@if-pan.krakow.pl (M.D.-W.); 2 Laboratory of Pharmacogenomics, Department of Molecular Neuropharmacology, Maj Institute of Pharmacology, Polish Academy of Sciences, Smętna Street 12, 31-343 Kraków, Poland; jacek.hajto@gmail.com (J.H.); marpiech@if-pan.krakow.pl (M.P.)

**Keywords:** miRNA, mRNA, NET-KO mice, SWR/J mice, restraint stress, stress resilience, stress susceptibility, signaling pathway, prefrontal cortex, next-generation sequencing

## Abstract

Three strains of mice with various susceptibilities to restraint stress (RS), i.e., mice with a knocked out norepinephrine transporter gene (NET-KO), SWR/J and C57BL/6J (WT) mice were shown to serve as a good model to study the molecular mechanisms underlying different stress-coping strategies. We identified 14 miRNAs that were altered by RS in the PFC of these mice in a genotype-dependent manner, where the most interesting was let-7e. Further in silico analysis of its potential targets allowed us to identify five mRNAs (Bcl2l11, Foxo1, Pik3r1, Gab1 and Map2k4), and their level alterations were experimentally confirmed. A next-generation sequencing (NGS) approach, which was employed to find transcripts differentially expressed in the PFC of NET-KO and WT mice, showed that, among others, two additional mRNAs were regulated by mmu-let-7e, i.e., mRNAs that encode Kmt2d and Inf2. Since an increase in Bcl2l11 and Pik3r1 mRNAs upon RS in the PFC of WT mice resulted from the decrease in mmu-let-7e and mmu-miR-484 regulations, we postulated that MAPK, FoxO and PI3K-Akt signaling pathways were associated with stress resilience, although via different, genotype-dependent regulation of various mRNAs by let-7e and miR-484. However, a higher level of Kmt2d mRNA (regulated by let-7e) that was found with NGS analysis in the PFC of NET-KO mice indicated that histone methylation was also important for stress resilience.

## 1. Introduction

Mood disorders, including major depressive disorder (MDD), are still a considerable burden for modern society. Despite the availability of approx. 50 different drugs, the treatment of MDD is far from ideal, nor is the understanding of the mechanism of action of antidepressant drugs that are administered repeatedly (which is necessary to achieve clinical efficacy). Most of the numerous behavioral, biochemical and molecular preclinical studies focused on various kinds of stress since it is regarded as the main factor contributing to mood disorders. Stress is defined as a condition that seriously perturbs the psychological and physiological balance of an individual. Serious stress, however, does not affect everyone in the same manner; some susceptible individuals (not only humans but also animals) adapt poorly to stressors and express inappropriate responses that can become persistent states of stress themselves. On the other hand, some resilient individuals can perceive adversity and develop adaptive psychological and physiological responses. The underlying mechanisms of these responses are not fully understood, although they are known to depend on a combination of genetic and non-genetic factors that interact in complex ways. Coping strategies are essential to minimize the impact of stress and determine the degree of resilience and susceptibility; however, as the biological basis of the stress response is not clearly defined, the same is true for coping strategies [1]. 

Stress susceptibility and resilience in pre-clinical rodent models are often studied in relation to MDD [2,3]. Three strains of mice with various susceptibility to restraint stress (RS), i.e., mice with a knocked-out gene that encodes the norepinephrine transporter (NET-KO), as well as C57BL/6J (WT) and SWR/J mice, were shown to serve as a good model to study the mechanisms underlying different stress-coping strategies. In our recent study, we were able to show alterations at the level of microRNAs (miRNAs) in the serum of these three genotypes, both between strains and regarding their response to RS [4,5]. MicroRNA molecules present in the blood may serve as potential biomarkers for brain pathologies, including depressive disorders [6,7], but their presence and possible alterations in the specific brain regions are even more interesting. In the present study, we chose the prefrontal cortex (PFC) since it is widely accepted that this brain region is involved in the adaptive control of behavior. Acute stress influences the activity of PFC, which in turn results in long-term neurobiological alterations [8]. The molecular mechanisms underlying these alterations, especially mechanisms that differentiate stress responses, namely, resilience and susceptibility, are not fully understood, but the increasing number of experimental data indicates that phenomena of epigenetic regulation of gene expression by microRNA have gained great interest. MicroRNA molecules, which are widely expressed in eukaryotes, are small (17–24 nucleotides), non-coding RNA transcripts that play an important role in the post-transcriptional regulation of many genes; therefore, studying alterations at miRNA level one can allow for gaining deeper insight into the complex network and mutual relationships of various transcripts that are targeted by a given miRNA. 

In the present study, we identified miRNAs that were differentially expressed in the PFC of three strains of mice, which were characterized earlier as displaying different stress-coping strategies. Additionally, we were able to identify miRNAs that were altered by RS in the PFC of these mice. Fourteen of them were altered upon RS in a genotype-dependent manner, where the most interesting was let-7e. Furthermore, using DIANA-miRPath v.3.0, an online software, the assessment of the defined miRNA regulatory roles and the identification of controlled pathways was carried out [9]. It allowed us to identify the transcripts that were targeted by identified miRNAs. Five mRNAs that were regulated by let-7e were altered upon RS in a genotype-dependent manner. The alterations in the levels of these transcripts in the mouse PFC was experimentally verified. We also employed a next-generation sequencing (NGS) approach to find additional transcripts that were differentially expressed in the PFC of NET KO and WT mice.

## 2. Results

### 2.1. miRNAs Expression Changes in the Prefrontal Cortex

**Figure 1:**
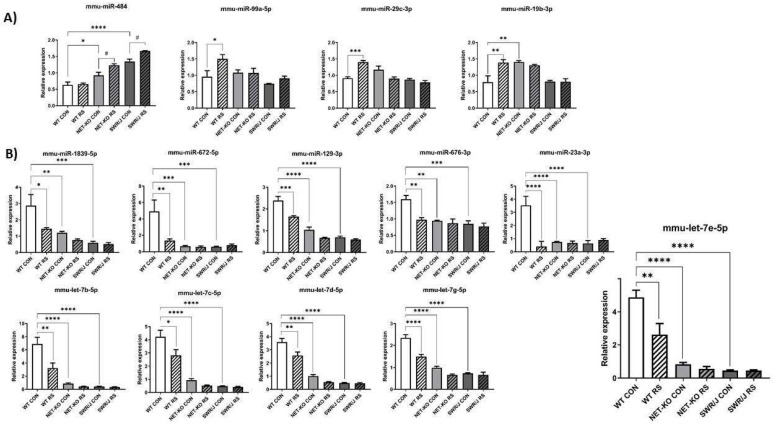
The expression of miRNAs in the prefrontal cortex (PFC) of three genotypes of mice following restraint stress (RS). (**A**,**B**) show the expression of miRNAs that were altered upon RS. WT—wild-type mice; NET-KO—norepinephrine transporter knock-out mice; SWR/J—Swiss mice. CON—control group; RS—restraint stress group. * *p* ≤ 0.05, ** *p* ≤ 0.01; *** *p* ≤ 0.001; **** *p* ≤ 0.0001; # *p* ≤ 0.05; *n* = 4.

The expression changes of miRNAs under a stress condition (RS) in the PFC were compared depending on the genotype. Special attention was paid to miRNAs, whose expression changed differently following RS in the PFC of NET-KO and SWR/J mice as compared to WT mice. Fourteen such miRNAs were discovered. The expression of four of them was upregulated upon RS (mmu-miR-484 (F (1,18) = 17.21; *p =* 0.0006); mmu-miR-99a-5p (F (1,18) = 5.969; *p =* 0.02); mmu-miR-29c-3p (F (1,17) = 13.27; *p =* 0.0003); mmu-miR-19b-3p (F (1,17) = 4.176; *p =* 0.05)). The expression level of mmu-miR-484 was upregulated in the PFC of NET-KO and SWR/J upon RS, while the level of mmu-miR-99a-5p, mmu-miR-29c-3p and mmu-miR-19b-3p was elevated in the PFC of WT animals following RS (Figure 1A). The expression of the remaining miRNAs was reduced in the PFC of NET-KO and SWR/J, both under control and stress conditions, as compared to the WT control animals (mmu-miR-1839-5p (F (1,18) = 7.519; *p =* 0.013); mmu-miR-672-5p (F (1,18) = 6.001; *p =* 0.02); mmu-miR-129-3p (F (1,16) = 30.01; *p* < 0.0001); mmu-miR-676-3p (F (1,17) = 11.87; *p =* 0.003); mmu-miR-23a-3p (F (1,17) = 16.68; *p =* 0.0008); mmu-let-7b-5p (F (1,18) = 10.3; *p =* 0.0049); mmu-let-7c-5p (F (1,18) = 7.788; *p =* 0.01); mmu-let-7d-5p (F (1,18) = 13.95; *p =* 0.0015); mmu-let-7g-5p (F (1,18) = 27.65; *p* < 0.0001); mmu-let-7e-5p (F (1,18) = 9.628; *p =* 0.006)). Likewise, the expression of these miRNAs was decreased upon RS in the PFC of WT mice (Figure 1B).

### 2.2. KEGG Pathway Analysis

**Figure 2:**
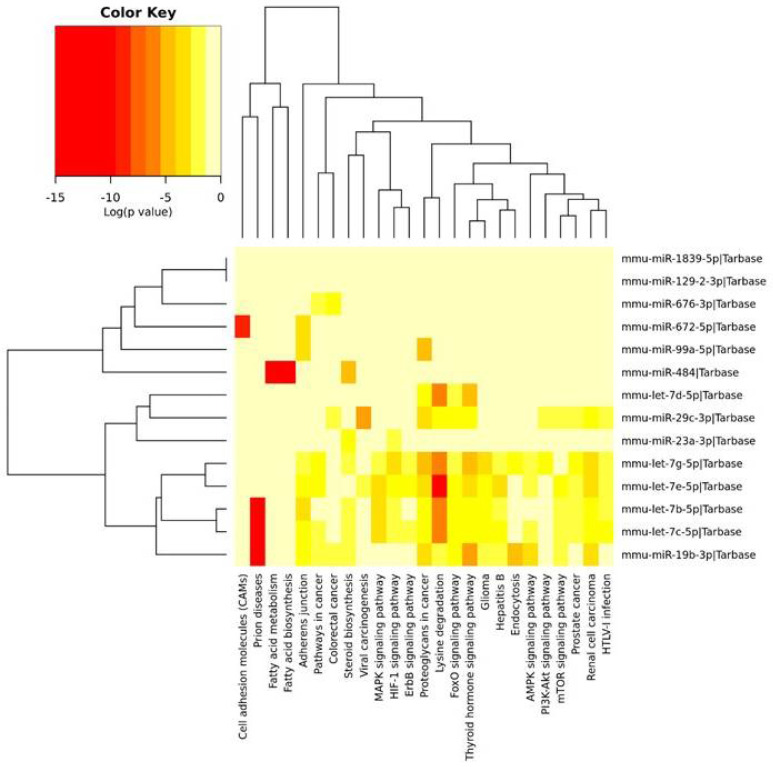
The heatmap indicating the associations of miRNAs with the KEGG pathways. The miRNAs are on the right-hand side, while the KEGG pathways are at the bottom of the map. The significances of linking miRNAs to the KEGG categories are color-coded (the scale in the upper-left corner).

The KEGG pathway analysis allowed us to find 25 pathways, which included mRNAs that were regulated by miRNAs identified in the PFC and described above. These miRNAs were clustered into three groups. The first cluster was associated with the fewest number of pathways, i.e., cell adhesion molecules (CAMs), fatty acid metabolism and biosynthesis, as well as the adherens junction and steroid biosynthesis. The second cluster, containing three miRNAs, was associated with lysine degradation and the thyroid hormone signaling pathway. The third cluster of miRNAs was associated with the regulation of the greatest number of pathways, i.e., lysine degradation, the adherens junction, the MAPK signaling pathway, thyroid hormone signaling pathway, endocytosis and the AMPK signaling pathway, as well as the mTOR and PI3K-Akt signaling pathways (Figure 2).

### 2.3. GO Annotation Analysis

**Figure 3:**
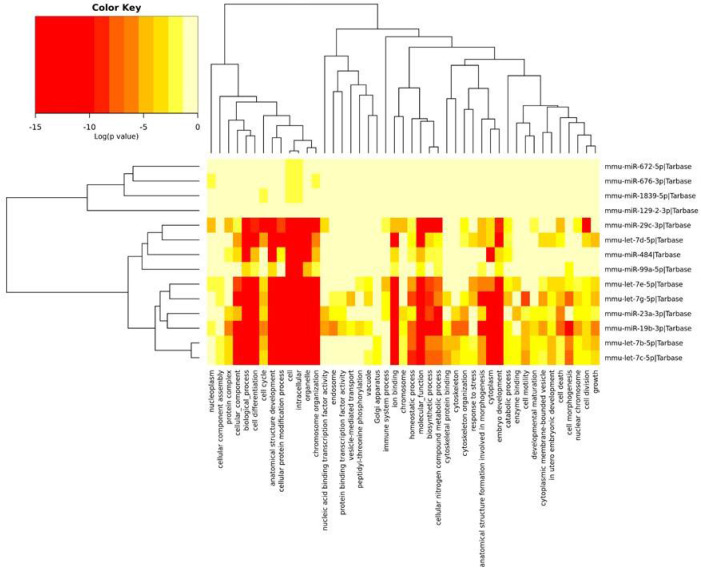
The heatmap indicating the associations of miRNAs with the GO annotations. The miRNAs are on the right-hand side, while the GO annotations are at the bottom of the map. The significances of linking miRNA to GO annotations are color-coded (the scale in the upper-left corner).

The miRNAs identified in the PFC and described above were divided into three groups depending on the Gene Ontology (GO) annotation. Two miRNA clusters were significantly associated with most of the annotations. Most miRNAs were associated with chromosome organization, ion binding, homeostatic processes and biosynthetic processes. The mmu-miR-19b-3p and mmu-miR-23a-3p miRNAs affected mRNAs encoding for the nucleic acid binding transcription factor and the protein binding transcription factor. In turn, miRNAs such as mmu-let-7c-5p, mmu-let-7b-5p, mmu-let-7g-5p, mmu-let-7d-5p and mmu-let-7e-5p were associated with the stress response. The remainder of miRNAs were also associated with the regulation of the immune system process (Figure 3).

### 2.4. mRNAs’ Expression Changes in the Prefrontal Cortex

The KEGG pathway analysis made it possible to select a group of mRNAs that were significantly regulated in the PFC. This group of mRNAs was compared to the mRNAs that were associated with the response to stress annotation via GO analysis. In addition, there were also some interesting mRNAs related to the response to stress annotation and a few selected from previous research (Supplement S1). Because the most interesting were those mRNAs that were regulated by mmu-let-7e-5p, they were further investigated. Twelve mRNAs were expressed in the PFC of all the studied mouse strains, while the expression of five of them changed in response to RS, depending on the genotype (Bcl2l11 (F (2,18) = 4.059; *p =* 0.035); Foxo1 (F (2,18) = 3.819; *p =* 0.0415); Pik3r1 (F (2,18) = 6.636; *p =* 0.0069); Gab1 (F (2,18) = 8.769; *p =* 0.0022); Map2k4 (F (2,18) = 7.803; *p =* 0.0036)). 

The expression of the mRNAs that encode Bcl2l11, Foxo1 and Pik3r1 increased following RS in the PFC of WT mice, while it remained unchanged in the NET-KO and SWR/J mice. Additionally, the expression of the mRNA that encodes Bcl2l11 was significantly higher in the PFC of the SWR/J control mice compared to the WT control group. 

The expression of the mRNAs that encode Gab1 and Map2k4 was decreased after RS in the PFC of WT mice, while it was significantly lower in the PFC of NET-KO and SWR/J mice as compared to the control group of WT mice and remained unchanged in these genotypes following RS.

**Figure 4:**
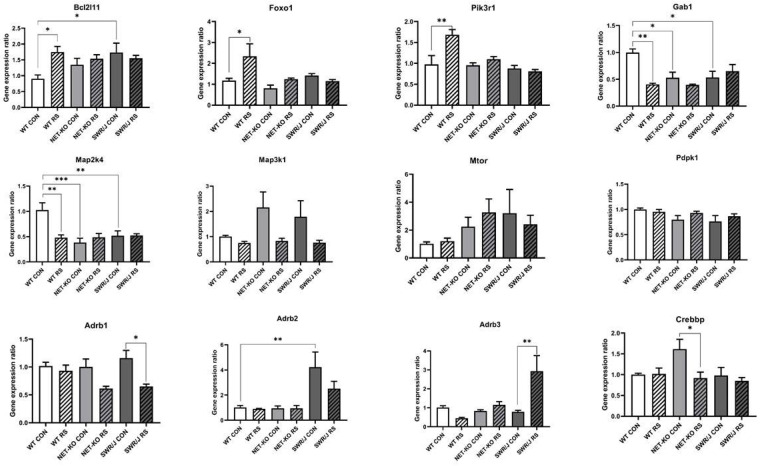
The expressions of mRNA-encoding genes that were regulated by mmu-let-7e-5p in the prefrontal cortex (PFC) of wild-type (WT), norepinephrine transporter knock-out (NET-KO) and Swiss (SWR/J) mice under the control condition (CON) and following stress (RS). Bcl2l11—BCL2-like 11 (apoptosis facilitator); Foxo1—forkhead box O1; Pik3r1—phosphoinositide-3-kinase regulatory subunit 1; Gab1—growth factor receptor-bound protein 2-associated protein 1; Map2k4—mitogen-activated protein kinase kinase 4; Map3k1—mitogen-activated protein kinase kinase kinase 1; Mtor—mechanistic target of rapamycin kinase; Pdpk1—3-phosphoinositide dependent protein kinase 1; Adrb1—adrenergic receptor, beta 1; Adrb2—adrenergic receptor, beta 2; Adrb3—adrenergic receptor, beta 3; Crebbp—CREB binding protein. * *p* ≤ 0.05, ** *p* ≤ 0.01; *** *p* ≤ 0.001; *n* = 4.

In turn, the expression of Adrb1 and Crebbp mRNAs decreased after RS in the PFC of SWR/J and NET-KO mice, respectively, while the expression of the mRNA that encodes Adrb3 increased in the PFC of SWR/J mice following RS, and the expression of Adrb2 mRNA was significantly higher in the PFC of SWR/J control group compared to WT control mice (Figure 4).

### 2.5. Correlation between miRNAs and mRNAs

To confirm the relationship between mmu-let-7e-5p and the mRNA changes following stress in a genotype-dependent manner, we performed bioinformatic analysis. The correlation was determined using the Pearson coefficient (r). 

The mmu-let-7e-5p showed a significant negative correlation with mRNA encoding Bcl2l11 and a positive correlation with Gab1 and Map2k4. Meanwhile, the positive correlation of this miRNA with Foxo1 and Pik3r1 was not significant (Table 1).

Additionally, we carried out an analysis to detect other interesting miRNAs that regulated mRNAs and were changed under stress conditions depending on the genotype. Therefore, the miRWalk platform was employed. The results of this analysis are presented in Supplement S2. The mmu-let-7b-5p, mmu-let-7c-5p, mmu-let-7d-5p, mmu-miR-129-3p, mmu-miR-23a-3p, mmu-miR-484, mmu-miR-672-5p and mmu-miR-676-3p miRNAs were among the miRNAs that regulated selected mRNAs. Therefore, further analysis of correlations was carried out between these miRNA and selected mRNAs. 

The mRNA encoding Bcl2l11 showed a significant negative correlation with almost all tested miRNAs, while Gab1 indicated a significant positive correlation with all miRNAs that regulate this mRNA. Likewise, the mRNA that encodes Map2k4 showed a significant positive correlation with almost all tested miRNAs, except mmu-miR-484, which showed a significant negative correlation with this mRNA. The mmu-miR-484 miRNA also indicated a significant negative correlation with the mRNA that encodes Pik3r1 (Table 1).

### 2.6. Next-Generation Sequencing of the Prefrontal Cortex

To find additional mRNAs that differentiated the PFC of NET-KO mice from WT mice or mRNAs that were regulated by mmu-let-7e-5p, we performed next-generation sequencing. After an adjustment for genetic-background-dependent differential expression, the final list of transcripts was identified. A total of 43 transcripts differentiated the PFC of NET-KO mice from WT mice (Supplement S3A). The over-representation of transcripts that encode peptides or their receptors (Cort, Gh, Npy, Vgf, Ntrk3) and those associated with long-term synaptic enhancement (LTP), namely Grin2b, Vamp2 and Shank1, was observed. The Pole and Kmt2d with exodeoxyribonuclease and methyltransferase activity, respectively, were also identified among these transcripts. The Zc3h7a gene was associated with miRNA binding and it also encoded transcription factors, as well as Pole and Npas4 (Supplement S3C,F). Considering the cellular location of transcripts in the PFC, Vgf, Npy, Shank1, Vamp2, Pclo, Cort, Bsn, Grin2b, Cdr1 and Npas4 were reported as being enriched in neurons, among other brain cells. The expression of Kmt2d, Ntrk3, Cd38 and Il33 was enriched in astrocytes; the expression of Spp1, Tpm3-rs7 and H2-Aa was enriched in microglia; and the expression of Plp1, Cnp, Nipal4, Mobp, Mal, Nectin1, Bfsp2 and Inf2 was enriched in newly formed oligodendrocytes and myelinating oligodendrocytes. Additionally, mRNAs that encode Mgp and Fn1 were found in endothelial cells (Supplement S3B). The pathways that were regulated by the identified mRNAs concerned the receptors’ interaction with neuroactive ligands or extracellular matrix and processes related to the functioning of the glutamatergic and dopaminergic synapses, as well as the PI3K-Akt and oxytocin signaling pathways. They were also associated with insulin secretion and circadian entrainment (Supplement S3D). The transcripts that differentiated the PFC of NET-KO mice from WT mice were involved in biological processes related to synapse and transport regulation. The Kmt2d gene was associated with the regulation of the intracellular steroid hormone receptor signaling pathway, while Spp1 was associated with the steroid catabolic process. Some of the genes, such as Tnrc18, Hecw2, Zc3h7a, Vgf, Spp1, Fn1 and Kmt2d, were involved in the epigenetic processes, and the Inf2 gene was associated with the regulation of mitochondrial fission (Supplement S3E).

**Figure 5:**
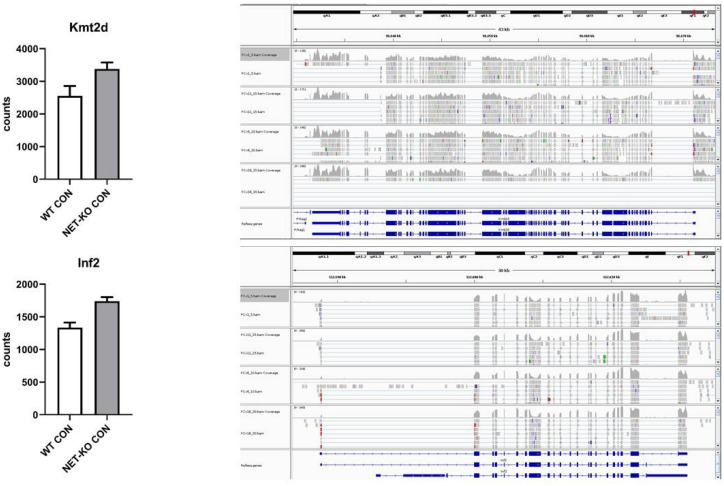
The expression of selected transcripts that were regulated by mmu-let-7e-5p in the prefrontal cortex of wild-type (WT) and norepinephrine transporter knock-out (NET-KO) mice under the control condition, as shown by NGS analysis. The graphs show the number of RNA-seq reads (Counts) overlapping a given gene. The right panels present the suitable RNA-seq tracks (two samples per group) that were inspected using the IGV viewer. The gene structures and annotations were obtained from the Ensembl database.

The mRNAs that encode Kmt2d and Inf2 were regulated by mmu-let-7e-5p and were over-represented in the PFC of NET-KO mice as compared to WT animals (Figure 5).

## 3. Discussion

In the previous study, we were able to identify numerous miRNAs that are present in the mouse serum, which differentiated three strains of mice depending on their response to restraint stress (RS) [4,5]. These miRNAs might be regarded as biomarkers of stress resilience. Our present study aimed to examine whether these miRNAs are also altered in the mouse brain. We chose the prefrontal cortex (PFC) as the brain area associated with stress and depression. Out of the 192 miRNAs that were selected based on our previous research, we found 14 that changed in the PFC upon RS, and these changes were dependent on the genotype: we observed a decrease in the level of the majority of identified miRNAs in the PFC of WT mice subjected to RS, and the lower level of the same miRNAs in the PFC of other two genotypes, which were regarded as resilient to RS. The stress-induced decrease of a majority of identified miRNAs in the PFC of mice subjected to RS can be regarded as an analogy to the results obtained by Smalheiser et al. [10], who showed a decreased level of miRNAs in the PFC of humans experiencing prolonged stressful conditions. Taken together, these results indicate that lower levels of certain miRNAs enable an organism to better cope with stress, probably via reduction of the main effect of miRNAs, i.e., the inhibition of translation processes. On the other hand, our previous studies [4,5] showed that the effect of RS was different in the mouse serum, i.e., more miRNAs were downregulated (10) than upregulated (4) in the PFC, while in the serum, the relations were the opposite (15 miRNAs were upregulated vs. 10 that were downregulated). Among these miRNAs were mmu-miR-99a-5p and mmu-miR-23a-3p, which were also detected in the PFC in the present study. 

In contrast, the expression of mmu-let-7b-5p, mmu-let 7c-5p and mmu-let-7g-5p decreased similarly in the blood and PFC of WT mice under stress conditions. Among the miRNAs identified in the PFC, some were also identified in the serum, and they were associated with stress resilience, e.g., mmu-miR-19b-3p, the expression of which increased upon RS, both in the PFC and serum of WT mice, and remained unchanged in NET-KO and SWR/J mice [4,5]. 

One of the miRNAs that were upregulated in the PFC of NET-KO and SWR/J mice compared to WT mice, namely, miR-484, was also increased in the PFC of these genotypes under stress conditions, indicating its involvement in stress resilience. As was shown by Wingo et al. [11] that predicted targets of miR-484 were enriched in the protein co-expression module involved in synaptic transmission and regulation of long-term synaptic plasticity. Wingo et al. [11] also found a significantly lower level of miR-484 in the PFC (obtained postmortem) of humans who had been subjected to a longitudinal assessment for late-life depressive symptoms. Such results may indicate that acute stress, such as RS in our studies, led to increased expression of miR-484, while long-term stressful conditions led to downregulation of this species. Additionally, this miRNA showed a negative correlation with Map2k4 and Pik3r1, which indicated that MAPK, FoxO and PI3K-Akt signaling pathways could be connected with stress resilience. Another miRNA that was increased in the PFC of WT mice following RS, namely, miR-29c, was associated with the effect of social defeat stress in mice [12] and also with the effect of stress and bipolar disorder in humans [13]. Similarly interesting is miR-19b, the expression of which was increased in the PFC of WT mice following RS. It was shown that this miRNA was increased in the amygdala of mice that were subjected to chronic mild stress, and it affected the regulation of mRNA encoding of the adrenergic receptor β-1 (Adrb1) [14]. In the present study, we showed a higher expression of miR-19b in the PFC of NET-KO mice, which can be regarded as a stress-resilient genotype. However, we did not find any alterations in the level of mRNA that encodes Adrb1. Nevertheless, it is worth noting that the level of adrenergic receptor β-1 protein was significantly lower in the PFC of NET-KO mice [15], which indicates that the relationship between the level of specific miRNA, its target mRNA and the protein encoded by this mRNA is not always straightforward.

It is worth noting that a decrease in mmu-miR-129-3p expression upon RS was found in the present study, similarly to findings provided by Buran et al. [16], who showed a decrease in this miRNA in the PFC of BALB/c mice subjected to chronic mild stress. On the other hand, we were able to find new stress-related miRNAs that varied with genotype and could be related to stress resilience. They included mmu-miR-1839-5p, mmu-miR-672-5p and mmu-miR-676-3p. 

An interesting similarity between the effects of RS on miRNAs in the serum and the PFC was the RS-induced downregulation of the whole group of miRNAs encoding the let-7 family—mmu-let-7b-5p, mmu-let-7c-5p, mmu-let-7d-5p, mmu-let-7g-5p and mmu-let-7e-5p in WT mice—and significantly lower levels of these miRNAs in the PFC of NET-KO and SWR/J mice, which were not altered by RS in these mice. The decrease in miR let-7b expression was described in the blood of students under exam-induced stress conditions [17], similarly to patients diagnosed with major depression, where the expression of miR let-7c was also decreased in the blood of these patients [18]. Recently, Maurel et al. [19] showed a decrease in mmu-let-7d-5p expression in the PFC of mice that were subjected to RS (24 h), however with no correlation with the level of its mRNA targets. 

Out of this miRNA family, let-7e seems to be the most interesting since its lower level was described in both the blood of major depressive disorder patients and in the PFC of the genetic rat model of depression, which is associated with an increase in a pro-inflammatory marker of depression (interleukin-6) [20,21]. The results obtained in the present study show that the expression of mmu-let-7e-5p decreased following RS in WT mice, and was lower and did not change following RS in the PFC of stress-resilient genotypes (NET-KO and SWR/J). 

Further in silico analysis of potential targets of mmu-let-7e-5p allowed us to identify mRNAs that encode for Bcl2l11, Foxo1, Pik3r1, Gab1 and Map2k4 genes in the PFC. The alterations in the level of the above-mentioned transcripts were experimentally confirmed. These transcripts are associated with FoxO (forkhead box O), PI3K-Akt (phosphoinositide-3-kinase–protein kinase B/Akt), AMPK (5’AMP-activated protein kinase), ErbB (tyrosine kinases receptor) and MAPK (mitogen-activated protein kinase) signaling pathways. These pathways seem to be differently regulated, which in turn can result in either susceptibility or resilience to stress. 

The results obtained in the present study are in line with other research that showed the involvement of these pathways in the stress response. Yang and coworkers demonstrated the association of PI3K-Akt and MAPK signaling pathways with resilience to psychological stress in the PFC of mice [22], and Liu et al. showed changes in Foxo1 expression under chronic unpredictable stress in the mouse PFC, which correlated with changes in Bdnf expression [23]. We also showed an RS-induced increase in the mRNA encoding Foxo1 in the PFC of WT mice, which correlated with the changes in the expression of Bdnf. The expression of Bdnf was increased following RS in the PFC of WT mice but did not change in the PFC of NET-KO and SWR/J mice (Supplement S4). This is in line with the studies by Maurel and coworkers, who discovered an increase in the expression of mRNA encoding Bdnf in the PFC of stress-susceptible mice and no change in stress-resilient mice [19]. 

On the other hand, the results obtained in the present study showed no change in mTOR mRNA expression that would be dependent on the genotype or effect of RS. Similarly, stress-independent changes in the expression of mRNA encoding mTOR in the PFC of immobilized rats were reported in other studies [24].

To find additional mRNAs that differentiate the PFC of NET-KO mice from WT mice or are regulated by mmu-let-7e-5p, aside from those that were already studied, we used the NGS approach. Out of the transcripts that differentiated these two genotypes, two mRNAs were regulated by mmu-let-7e, i.e., mRNAs encoding Kmt2d and Inf2. It is worth noting that a higher expression of these mRNAs in the PFC of NET-KO mice negatively correlated with the expression of mmu-let-7e. Kmt2d points to histone methyltransferase activity specific for H3-K4, and it is associated with histone lysine methylation. The appropriate correction of epigenetic factors related to environmental differences in cognitive abilities requires determining the mechanisms of chromatin modifications and variations in DNA methylation. Transposons representing stress-sensitive DNA elements appeared to mediate the environmental influence on epigenetic modifications [25]. This is interesting in the context of data showing that increased expression of the stress-responsive genes results from a lack of histone H3 lysine (K) 4 methylation [26]. Thus, an increased expression of mRNA encoding Kmt2d in the PFC of NET-KO mice can be associated with the stress resilience of these animals. On the other hand, the second mRNA, which encodes inverted formin-2 (Inf2), instead acts at the cellular level and it was shown that its expression changes under oxidative stress [27]. 

Additionally, we found that the level of transcripts that encode some neuropeptides and their receptors was higher in the PFC of NET-KO mice as compared to WT animals, which is especially interesting since Chmelova and colleagues [28] showed that rats isolation induced reduction in Vgf (VGF nerve growth factor inducible) gene expression and Farhang at al. [29] provided data indicating that the expression mRNA encoding Ntrk3 (neurotrophic tyrosine kinase, receptor, type 3) was significantly higher in the stress-resilient rats. Likewise, a higher level of cortistatin (Cort) decreased the expression of Pomc mRNA (encoding ACTH) in mice, which silenced the HPA (hypothalamic-pituitary-adrenal) axis [30]. Additionally, the expression of mRNA encoding Npy (neuropeptide Y) was increased after chronic mild stress in the PFC of females but not the males [31]. These studies indicate that the higher expression of mRNAs encoding neuropeptides and their receptors in the PFC of NET-KO mice might be related to their stress resilience. 

Interestingly, the PI3K-Akt signaling pathway, which is described above as being regulated by mmu-let-7e-5p, was regulated by three (Gh, Spp1, Fn1) of the mRNAs that differed in the PFC of NET-KO mice relative to WT mice. This pathway was shown above to be related to the stress response. Therefore, one can assume that its regulation is associated with stress resilience.

Additionally, an increase in mRNAs that encode Bcl2l11 upon RS in the PFC of WT mice resulted from a decrease in mmu-let-7e, while the Pik3r1 decrease resulted from mmu-miR-484 regulations. Therefore, we postulate that mainly MAPK, FoxO and PI3K-Akt signaling pathways are associated with stress resilience, although via different, genotype-dependent regulation of various mRNAs via let-7e and miR-484. However, the differences in gene expression (Kmt2d) regulated by let-7e that were found with NGS analysis indicated that histone methylation was also important for stress resilience.

## 4. Materials and Methods

### 4.1. Animals

The mice were generated by Xu and co-workers at Duke University Medical Center, Durham, NC, USA [32], and were obtained from Dr. M. Caron. Heterozygous mice (C57BL/6J background) were mated to each other to generate a homozygous WT, as well as NET-KO mice (Slc6a2tm1Mca/IFPAS); males, ca. 4.5 months old, were used for the experiments. The genotype of each mouse was confirmed with a polymerase chain reaction (PCR) using the following primers: mNETEx2s (5′-GCT TTA TGG CAT GTA GTG TGC AC-3′), mNETEx2as (5′-GCT TTC TGC TTG AAC TTG AAG GC-3′) and EGFPas (5′-GCC GGA CAC GCT GAA CTT GTG-3′) to amplify 700 and 500 bp PCR products from WT and NET-KO mice, respectively. The Swiss (SWR/J) strain was obtained from the Jackson Laboratory (USA). Six-week-old males and females were imported to IFPAS and mated to each other. The 4.5-month-old males were used for the experiments. The mice were housed in groups. Animals had free access to food and water and were kept at a constant room temperature (24 °C) under a 12 h light/dark cycle. The experiments were carried out according to the decision of the Minister of Environment (no. 156/2019) and the 2nd Local Institutional Animal Care and Use Committee (IACUC) (199/2017).

### 4.2. Restraint Stress (RS) and Prefrontal Cortex Collection

The mice were housed individually one week before the experiment. After the adaptation period, they were placed in well-ventilated polypropylene tubes (length 110 mm and diameter 28 mm) that were located in their home cage for 4 h. While being immobilized, the mice did not have access to food and water. Ten minutes after the RS procedure, the mice were sacrificed, and the brains were collected from 8 male mice per group, as well as from 8 mice that were not subjected to RS (control groups). The prefrontal cortex (PFC) was dissected medially (Bregma 3.08 mm to Bregma 2.1 mm) according to *The Mouse Brain in Stereotaxic Coordinates* [33] and stored at −80 °C until further purification.

### 4.3. Isolation of microRNA from the PFC

Total RNAs, including miRNAs, were isolated from the PFC of 4 mice per group. The miRNeasy Mini Kit (Qiagen, Germantown, MD, USA) was used for miRNA purification. These kits were selected according to Brown’s earlier research [34]. The procedures were performed according to the manufacturer’s instructions. A total of 700 µL of QIAzol Lysis Reagent was added to each sample, which was then homogenized 2 times for 2 min at 30 Hz. During the procedure, 140 µL of chloroform and 525 µL of 100% ethanol were added. A total of 40 µL of RNase-free water was used for elution RNAs of PFC samples. The quality and quantity of the isolated total RNA were evaluated using a NanoDrop ND-1000 (Thermo Fisher Scientific, Waltham, MA, USA).

### 4.4. miRNA RT-qPCR Array

Custom TaqMan Array MicroRNA Cards were used to determine the expression level of miRNAs. A total of 192 primers were selected according to our previous research on miRNAs that were expressed in the mouse serum [4]. One Custom TaqMan Array MicroRNA Card was used per sample in duplicate. The cDNA was synthesized using a TaqMan MicroRNA Reverse Transcription Kit (Thermo Fisher Scientific, Waltham, MA, USA) with a TaqMan Custom RT Pool according to the manufacturer’s instructions. The obtained cDNA was mixed with TaqMan Universal PCR Master Mix, No AmpErase UNG (Thermo Fisher Scientific, Waltham, MA, USA) to perform the RT-qPCR reactions. The RT-qPCRs were running on a QuantStudio 12K Flex System (Applied Biosystems, Waltham, MA, USA). Data were further analyzed with QuantStudio 12K Flex software (Applied Biosystems, Waltham, MA, USA). Ct values above 34 were considered to represent undetectable miRNAs. The same threshold equal to 0.2 was set for all samples for comparison. Then, the data were analyzed with qBasePLUS 3.1 software (Biogazelle, Zwijnaarde, Belgium), which enables whole-genome miRNA profiling with global mean normalization. The miRNAs that were suitable for normalization, namely, mmu-miR-9-3p and mmu-miR-30c-5p, were generated using the geNorm algorithm [35,36]. Afterward, statistical analysis was carried out with GraphPad Prism 8.4.2 using two-way ANOVA (Tukey’s post hoc test for multiple comparisons between groups) to compare each miRNA expression change between genotypes under control conditions and following RS. A value of *p* ≤ 0.05 was considered to be significant.

### 4.5. Identification of miRNA targets In Silico

The miRNAs with expression levels that significantly differentiated the stress response depending on the mouse strain were subjected to further bioinformatics analyses. DIANA-miRPath v.3.0, an online software, was used for the assessment of miRNA regulatory roles and the identification of controlled pathways [9]. Following pathway enrichment analysis using Fisher’s exact test (hypergeometric distribution), the miRNAs targets were selected from TarBase v7.0. A value of *p* ≤ 0.05 was considered to be significant and FDR correction was selected. Then, the results of the computational approach were validated experimentally at the mRNA level.

### 4.6. Isolation of mRNAs from the PFC

The RNeasy Plus Mini Kit (Qiagen, Germantown, MD, USA) was used for isolating mRNA from the PFC according to the manufacturer’s instructions. The PFC samples (4 per group) were homogenized with 350 µL of the buffer RTL Plus for two times of 2 min at 40 Hz with TissueLyser LT (Qiagen, Germantown, MD, USA). The same volume of 70% ethanol was added to each sample, while 40 µL of RNase-free water was added to the column for the RNAs’ elution from the PFC samples. The quality and quantity of the isolated total RNA were evaluated using a NanoDrop ND-1000 (Thermo Fisher Scientific, Waltham, MA, USA) and Experion Microcapillary Electrophoresis System (Bio-Rad, Hercules, CA, USA). Samples that passed the quality threshold (RIN > 8.0) were used for further experiments.

### 4.7. mRNA RT-qPCR Array

The Custom TaqMan Gene Expression Array Cards were used to determine the expression level of the mRNAs. The 32 primers that were placed on the array cards were selected mainly based on in silico analyses (Supplement S1). The mRNAs expression of four samples in triplicates was analyzed using one array card. The cDNA transcripts for RT-qPCR reactions were synthesized according to the manufacturer’s protocol using a High Capacity cDNA Reverse Transcription Kit (Thermo Fisher Scientific, Waltham, MA, USA). The obtained cDNA was mixed with TaqMan Universal PCR Master Mix, No AmpErase UNG (Thermo Fisher Scientific, Waltham, MA, USA) to perform the RT-qPCR reactions. The RT-qPCRs were run on a QuantStudio 12K Flex System (Applied Biosystems, Waltham, MA, USA). Data were further analyzed with the QuantStudio 12K Flex software (Applied Biosystems, Waltham, MA, USA). Ct values above 34 were considered to represent undetectable mRNAs. The same threshold equal to 0.2 was set for all samples for comparison. Then, the data of selected mRNAs were analyzed with a modified Pfaffl equation for multiple reference genes [37]. The beta-actin (Actb) and glyceraldehyde-3-phosphate dehydrogenase (Gapdh) were selected for normalization. Afterward, statistical analysis was carried out with GraphPad Prism 8.4.2 ( GraphPad Software, San Diego, CA, USA) using two-way ANOVA (Tukey’s post hoc test for multiple comparisons between groups) to compare each mRNA expression change between genotypes under control conditions and following RS. A value of *p* ≤ 0.05 was considered to be significant.

### 4.8. Bioinformatic Analysis of the Correlation between miRNAs and mRNAs

GraphPad Prism 8.4.2 was used to determine the Pearson coefficient (r) between the miRNAs and mRNAs that changed following restraint stress in a genotype-dependent manner. Additionally, the miRWalk v.3.0 platform was selected to determine the miRNAs that regulated interesting mRNAs [38]. This is a platform that uses a machine-learning-based algorithm, namely, TarPmiR, to predict miRNA–target interactions [39]. Otherwise, the miRWalk framework covers datasets of TargetScan v.7.1, miRDB v.5.0 and validated information from miRTarBase v.7.0 [40,41,42]. The appropriate species, 3’UTR region and *p*-value equal to 0.9 were selected from the degree filter during analysis.

### 4.9. Isolation of RNAs from the Prefrontal Cortex for the Next-Generation Sequencing Procedure

To analyze changes in the RNA expression of the PFC of NET-KO mice in comparison to WT mice under control conditions, tissue from 10 males (ca. 4.5 months old) in each group was collected. The RNeasy Plus Mini Kit (Qiagen, Germantown, MD, USA) was used with modification for total RNA purification according to the manufacturer’s instructions. The homogenization of samples was performed with 600 µL of RTL Plus buffer for 2 min at 40 Hz (two times) with TissueLyser LT (Qiagen, Germantown, MD, USA). The same volume of 70% ethanol was added to each sample, while 50 µL of RNase-free water was added to the column to elute the RNAs. During the procedure, the RNase-Free DNase Set (Qiagen, Germantown, MD, USA) was used to complete the DNA removal. A 10 µL DNase I stock solution mixed with 70 µL RDD buffer was added to each column. The quality and quantity of the isolated total RNA were evaluated using a NanoDrop ND-1000 (Thermo Fisher Scientific, Waltham, MA, USA) and an Experion Microcapillary Electrophoresis System (Bio-Rad, Hercules, CA, USA). Samples that passed the quality threshold (RIN > 8.0) were combined in groups of 5, and RNA sequencing was performed.

### 4.10. RNA Next Generation Sequencing Procedure

The Ribo-Zero rRNA Removal Kit (Epicentre, Madison, WI, USA) was used to remove ribosomal RNA. RNA libraries were prepared using the rRNA-depleted RNA with the NEBNext Ultra Directional RNA Library Prep Kit from Illumina (NEB, USA). Library sequencing was performed (150 bp paired-end, 40 M reads per sample) on an Illumina HiSeq platform (Illumina Inc., San Diego, CA, USA) as an external service (Novogene, Hong Kong). The quality check of the raw RNA-seq data was performed with fastQC v0.11.5 in R. Reads were aligned to the Mm10 mouse reference genome (GRCm38) from the Ensembl database using Tophat 2. Transcript counts were normalized to fragments per kilobase million fragments mapped (FPKM) with the Cufflinks v2 package [43]. The quality check of the raw RNA-seq data was performed with fastQC v0.11.5. Reads were aligned to the Mm10 mouse reference genome with annotations obtained from the Ensembl 90 database using Tophat2 v2.1.0. Transcript counts were normalized to fragments per kilobase million fragments mapped (FPKM) with the Cufflinks v2 package [43].

#### 4.10.1. The Statistical Analysis of the Next-Generation Sequencing Data

All samples were quality controlled using fastQC and aligned to the mouse reference genome (mm10 from the Ensembl database) with hisat2. The featureCounts tool, along with the gene annotation in gff3 format from the Ensembl database, were used for gene-level read summarization. Differential expression between the two genotypes was facilitated by the edgeR R package with the subsequent FDR *p*-value correction method. All statistical analyses were performed with R software v3.4. All analysis code is available in the project’s code repository (https://github.com/ippas/ifpan-solich-mouseko, accessed on 28 July 2021). Genes that differentiated NET-KO from WT mice in the PFC were subjected to analyses in Brain RNA-Seq (analysis of cellular localization of transcripts in the brain of mice) [44], EnrichR (functional analysis of overrepresentation of biological terms) [45] and TcoF-DB (database of known transcription factors) [46].

#### 4.10.2. Adjustment for Genetic-Background-Dependent Differential Expression

The list of genes differentiating NET-KO and WT mice in the PFC was subjected to the filtration of strain-specific SNPs. Transcripts were visualized in Integrated Genomics Viewer (IGV) [47], and genes with differences in SNPs between WT and NET-KO mice were identified. The strain-specific SNPs (i.e., C57Bl and 129SvJ) in the Strain and SNP database were retrieved from the Mouse Genome Database (MGD; Mouse Genome Informatics, The Jackson Laboratory, Bar Harbor, Maine; http://www.informatics.jax.org, accessed on 1 December 2018). Transcripts with 129Sv-specific SNPs were considered as being influenced by the transfer of alleles that were adjacent to the transgene and their altered expression resulted from the genetic background of NET-KO mice. 

## Figures and Tables

**Table 1:** Correlation matrix of miRNAs and mRNAs expression changing following stress in a genotype-dependent manner.

	Bcl2l11	Foxo1	Gab1	Pik3r1	Map2k4
mmu-let-7e-5p	**−0.43 (0.036)**	0.21 (0.327)	**0.49 (0.014)**	0.37 (0.071)	**0.70 (0.0002)**
mmu-let-7b-5p	**−0.43 (0.035)**	─	─	0.23 (0.283)	**0.59 (0.002)**
mmu-let-7c-5p	**−0.41 (0.048)**	─	**0.47 (0.019)**	─	**0.69 (0.0002)**
mmu-let-7d-5p	─	0.23 (0.281)	**0.47 (0.018)**	─	**0.65 (0.0005)**
mmu-miR-129-2-3p	**−0.45 (0.033)**	─	**0.55 (0.008)**	0.41 (0.060)	─
mmu-miR-23a-3p	**−0.54 (0.007)**	−0.06 (0.774)	─	−0.31 (0.155)	─
mmu-miR-484	0.38 (0.064)	─	─	**−0.53 (0.007)**	**−0.42 (0.038)**
mmu-miR-672-5p	**−0.41 (0.047)**	─	─	0.02 (0.913)	**0.59 (0.002)**
mmu-miR-676-3p	─	─	─	0.20 (0.348)	**0.68 (0.0003)**

Pearson coefficient (r) and *p*-values (between brackets) are shown in the table. Bold-type values are statistically significant.

## Data Availability

All analysis codes for the NGS are available in the project’s code repository (https://github.com/ippas/ifpan-solich-mouseko, accessed on 28 July 2021).

## Supplementary Materials

The following are available online at www.mdpi.com/article/10.3390/ijms22179439/s1, Table S1: The list of mRNAs regulated by miRNAs that were altered under restraint stress (RS) depending on the genotype. Table S2: The relationship of miRNAs that differentiated a stress-resistant genotype from a stress-susceptible genotype and their targets (mRNAs). Table S3: The results of an in silico analysis of the data obtained using NGS analysis. Figure S1: The expression of mRNA encoding Bdnf in the prefrontal cortex (PFC) of wild-type (WT), norepinephrine transporter knock-out (NET-KO) and Swiss (SWR/J) mice under the control condition (CON) and following stress (RS).
