# Supplementary figures and images for: MicroRNA Let-7e in the Mouse Prefrontal Cortex Differentiates Restraint-Stress-Resilient Genotypes from Susceptible Genotype
Source: Int J Mol Sci. 2021 Aug 30;22(17):9439. doi: 10.3390/ijms22179439 (PMC8430919; doi:10.3390/ijms22179439)

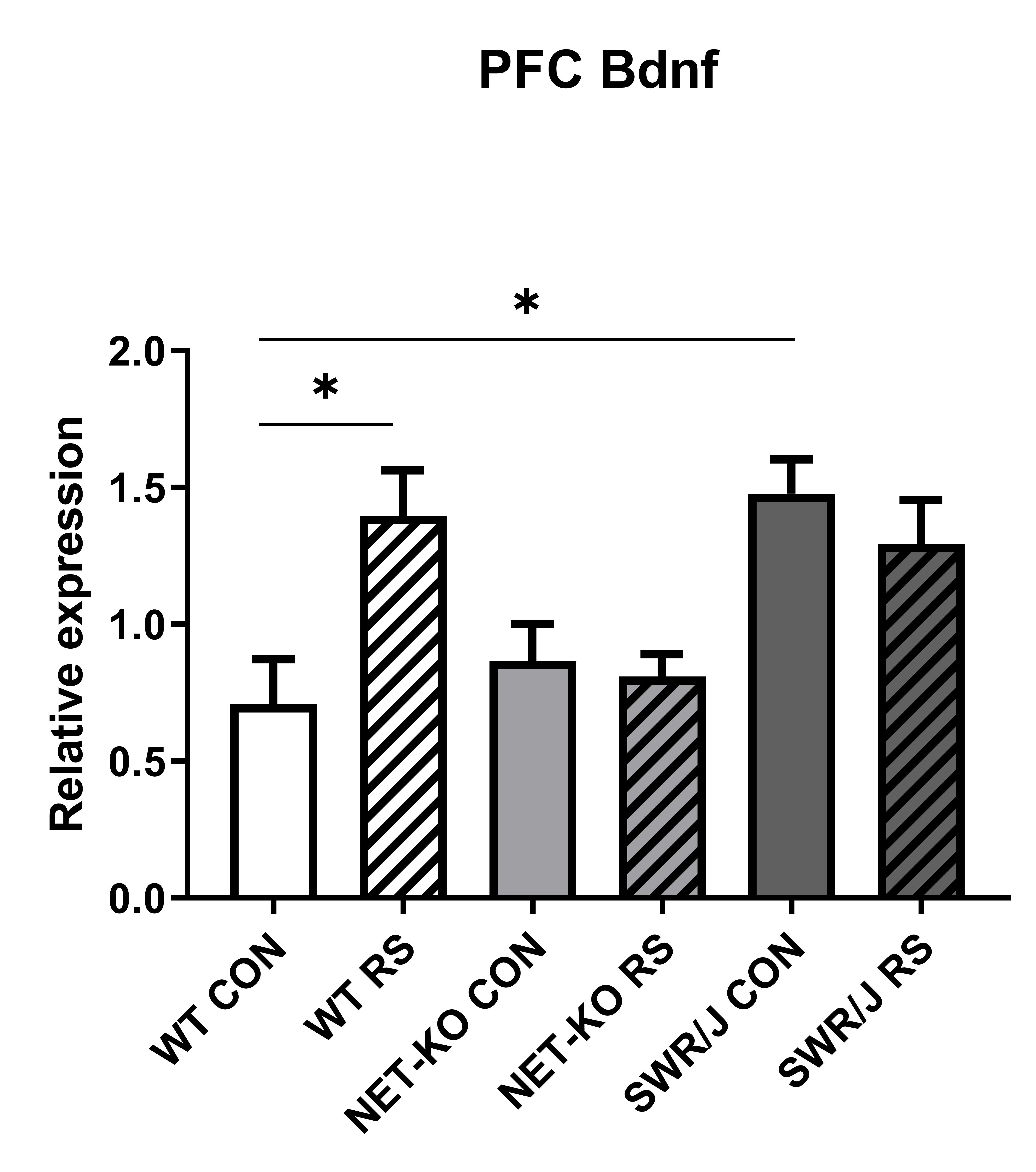

Supplement: Supplementary file 1 [file ijms-22-09439-s001.zip › Suppl Solich et al 28_07_2021/Suppl S4 PFC Bdnf.jpg]
